# Head and Neck Cancer Patients' Quality of Life: A Bibliometric Analysis Using Network Visualization Mapping

**DOI:** 10.1055/s-0045-1809665

**Published:** 2025-09-19

**Authors:** Sujal Parkar, Abhishek Sharma

**Affiliations:** 1 Department of Public Health Dentistry, Government Dental College and Hospital, Ahmedabad, Gujarat, India; 2 Department of Public Health Dentistry, Government Dental Collage and Hospital, (RUHS, College of Dental Sciences), Jaipur, Rajasthan, India

**Keywords:** bibliometric analysis, head and neck cancer, network, quality of life

## Abstract

**Introduction:**

The quality of life of head and neck cancer patients is significantly impacted. Bibliometric analysis is crucial to get the scholarly landscape, figuring out the research area related to quality of life among head and neck cancer patients.

**Objective:**

To use network visualization mapping to perform a bibliometric analysis of the quality of life among head and neck cancer patients.

**Methods:**

A literature search was done using the Scopus database. The searched keywords among papers included the amalgamation of *quality of life* , and *head and neck cancer* . The data were extracted, and bibliometric analysis was performed based on the bibliometric indicators: the trend of article publishing, citations, leading countries, and institutions contributing to a publication, potential authors, journals, and frequently occurring keywords. Network visualization mapping was performed using the VOSviewer software (Leiden University).

**Results:**

A total of 366 articles met the predefined eligibility criteria and were selected for analysis. The selected papers were published in 57 journals between 1983 and 2024. The results provide insightful information on there being a maximum of 25 articles in 2022 and the highest citation count of 1,683 in 2001. Among 52 countries, the United States was the leading contributor, having published 75 articles. The top contributing institution was Liverpool University Hospital, Aintree, United Kingdom, with four articles. Roger Simon was the author with the most potential, and *Head and Neck* was an impactful journal. The most frequently occurring keywords were *quality of life* , *head and neck* , and *oral cancer* .

**Conclusion:**

The current bibliometric analysis identified the scholarly impact and characteristics of articles, which provide researchers and policymakers with baseline data to frame research strategies for improving quality of life among head and neck cancer patients.

## Introduction

Globally, head and neck cancers (HNCs) constitute the 6th most common type of cancer diagnosed, with an annual incidence and mortality of 582,503 and 281,645, respectively. 1 The increased consumption of tobacco in both forms (smoking and non-smoking) and alcohol are the main culprits leading to HNC. 2 Viral infections such as human papillomavirus and Epstein bar virus (EBV) infections, chronic dental trauma, change in oncogenes, and tumor suppression genes are proved to be risk factors for developing HNCs. 3 Head and neck cancers arise from the head and neck region, encompassing the oral cavity, nose and paranasal air sinuses, larynx, and pharynx. These areas have intricated structural relationships and are necessary for vital functions like swallowing, chewing, and speaking. Hence, HNC patients are often given priority for special care over patients with other cancers. 4 Surgery, radiation, chemotherapy, and/or a combination of these treatments are the available options for treating HNC. Treatment-related side effects such as xerostomia, alternation in taste, difficulty in eating and swallowing, pain, fatigue, change in physical appearance, permanent disfigurement, and infirmity are unavoidable, even with the latest technological advancements and improvements in curative oncology. These treatment-related side effects have a substantial impact on quality of life (QoL). 5

The QoL concept encompasses a broad range of issues, including physical functioning, emotional wellbeing, and social functioning in which an individual lives. Patients suffering from HNC have to face devastating effects such as uncertainties about their recovery and fear of recurrence combined with various social, physical, financial, and emotional issues. 6 These issues can affect eating, drinking, speech, social appearance, and other day-to-day life functions, ultimately affecting patients' QoL. 7 8 Early detection of HNC amenable to single modality therapy offers the best prognosis, and, hence, the chance of long-term survival increases. The end point of this leads to improved QoL in the later stage of the patient's life. 5 Physical and mental distress, like depression and anxiety, along with factors such social stigma and lack of support can contribute to suicidal tendency among HNC survivors. The odds of suicide are significantly higher among HNC survivors compared with the general population. 9 With the evolving interest in patient-centered outcomes, assessing QoL has become essential to cancer research and clinical practice. 10

There is an abrupt rise in the scientific literature on QoL among HNC patients. Various studies on the development and validation of assessment tools for QoL, descriptive studies on QoL among HNC patients, the impact of cancer therapy on QoL, and predictors of QoL have been reported. Given the substantial growth of the already vast literature on this topic, staying current with every published article is a demanding task. To understand the focus and progression of research in this field, a bibliometric analysis (BA) provides a valuable tool for mapping the scholarly landscape, identifying key themes, and highlighting research gaps. Bibliometric analyses engage different tools such as maps, graphs, and network diagrams to visualize the results, making it easy to interpret them. It offers an organized examination of a substantial amount of data, facilitating the identification of patterns throughout time, study themes, changes in disciplinary boundaries, highly productive academics and institutions, and the general terrain of current research. 11 For this reason, assessing the QoL of HNC patients is crucial to getting a thorough picture, figuring out where the area needs further research, and organizing future contributions. 12

The literature review revealed that only one BA was conducted on QoL for HNC patients, by Ghazali SNA et al. 13 To the best of our knowledge, this is the second BA focusing on QoL among HNC patients. Hence, the current study was a bibliometric analysis that used network visualization mapping of the QoL among HNC patients to identify the present state of knowledge in this field and to pinpoint the research gaps that need to be conveyed in terms of trends in publications, the most potential researchers, the top articles and productive journals, countries, and research institutions, as well as frequently occurring keywords. By employing systematic and quantifiable methods, this study uncovered contributions to the field, influential studies, and potential areas for further research.

## Methods

The present BA was conducted in three phases: Phase 1: literature search and data collection, Phase 2: data extraction, and Phase 3: data and network visualization analysis.

### Phase 1: Literature Search and Data Collection

**Fig. 1:**
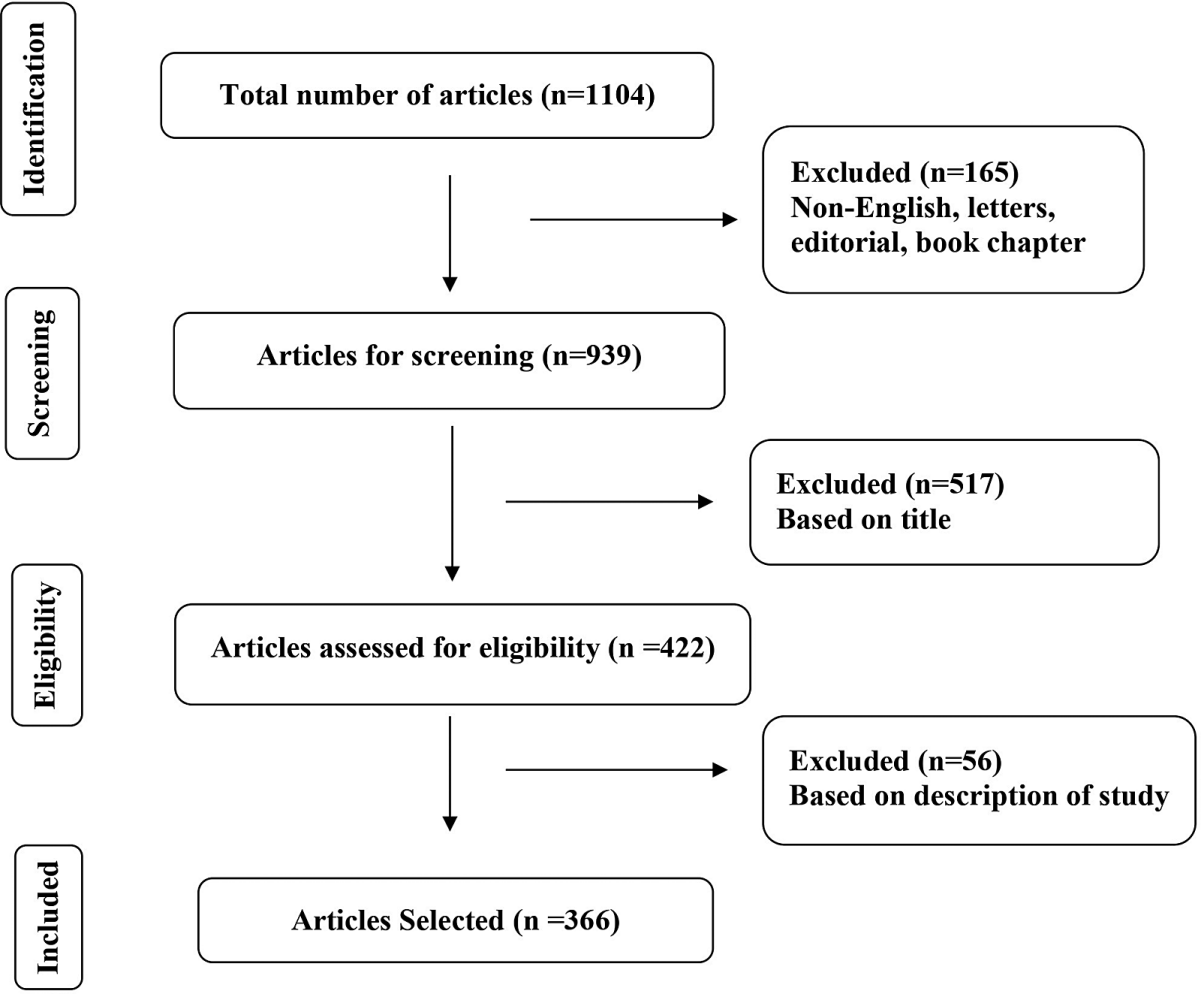
Preferred Reporting Items for Systematic Reviews and Meta-Analyses (PRISMA) flowchart for article selection.

The literature was searched using the Scopus electronic database in June 2024. Using the Scopus database, the search string was framed by using the keywords, *quality of life* , and *head and neck cancer* . The search string in Scopus database was: (TITLE ( *Quality of life* ) OR TITLE ( *Health-related quality of life* ) AND TITLE ( *Head and neck cancer* ) OR TITLE ( *Head and neck carcinoma* ) OR TITLE ( *Head and neck squamous cell carcinoma* ) OR TITLE ( *Oral Cancer* ) OR TITLE ( *Oral squamous cell carcinoma* ) OR TITLE ( *Oropharyngeal cancer* ) OR TITLE ( *Laryngeal cancer* )). No time restrictions were placed on the selection of the articles. Inclusion criteria: articles published in English language journals. Exclusion criteria: 1) conference papers, posters, letters, and pre-published articles; 2) clinical trials on chemotherapeutic drugs or radiation therapy procedures; 3) articles on recurrent tumors, metastases, or any systemic co-morbidity conditions; 4) articles on oral consequences following cancer therapy, such as xerostomia, mucositis, and disfigurement; and 5) articles on surgical reconstruction and dental rehabilitations. The articles were selected as per the guidelines of the Preferred Reporting Items for Systematic Reviews and Meta-Analyses (PRISMA) statement, 14 as depicted in Fig. 1 .

### Phase 2: Data Extraction

Both authors independently searched the papers through electronic searches. The two authors conducted a full-text appraisal based on the criteria, compared, and discussed the selected articles' eligibility, and made a final determination. Kappa statistics were used to identify the inter-rater agreement between the two authors.

Data such as the name of authors, the title of the article, publication year, author's affiliation, type of study, name of journals, institution, and country of the published article, number of citations of the article, author's keywords, and references were downloaded in Microsoft Excel (Microsoft Corporation) in comma-separated value (CVS) format.

### Phase 3: Data and Network Visualization Analysis

All extracted data was imported into Microsoft Excel 2019 (Microsoft Corporation). Microsoft Excel was used for descriptive statistics. The rank, frequency, averages, and graphs on the trends of publications and citations, and country-wise citation of articles were created by Microsoft Excel 2019. Mapchart, an online platform ( https://www.mapchart.net/world.html ), was used to display the country-wise distribution of the articles. VOSviewer software, version 1.6.18 (Leiden University), 15 was used for network mapping. The networks of most potential authors, top research articles and impactful journals, countries, and research institutions relied on the average citations of articles. The co-occurrence network for keywords was analyzed, based on the mean citations of articles having authors' keywords as a unit of analysis. Synonymous keywords were checked and merged to form a common keyword for the analysis, for example, *Quality-of-life* , *Quality* *of life* , *QoL* ; *EORTC-QLQ H&N35* , and *EORTC QLQ HN35* . Other keywords used were *head-neck cancer* and *head and neck cancer* .

Network visualization is presented as items, nodes, clusters, links, and total link strength. Each colored circle's size corresponds to the total number of articles contributed by authors, institutions, journals, as well as frequently occurring keywords. The number of articles from two authors, countries, institutions, or keywords is inversely proportional to the distance between the two circles. The relationship between authors, countries, institutes, journals, and keywords is represented by the lines joining the circle. Compared with those with warm colors, nodes with cool colors indicate fewer average citations.

## Results

A total of 1,104 articles were retrieved from Scopus electronic database. Out of 1,104 articles, 165 were not written in English, letters, editorial, pre-published, and published as book chapters, and hence they are excluded. The screening of titles led to the elimination of 517 articles from the remaining 939 articles. Further 56 articles were excluded based on the description of the study, yielding 366 articles for the final analysis. The inter-rater agreement between the 2 authors was strong, with a κ value of 0.91.

### Overview of Selected Articles

**Fig. 2:**
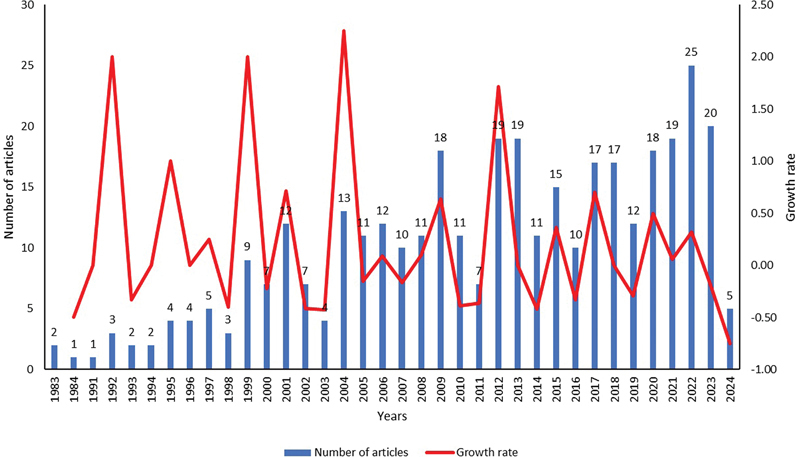
Trends of articles published.

**Fig. 3:**
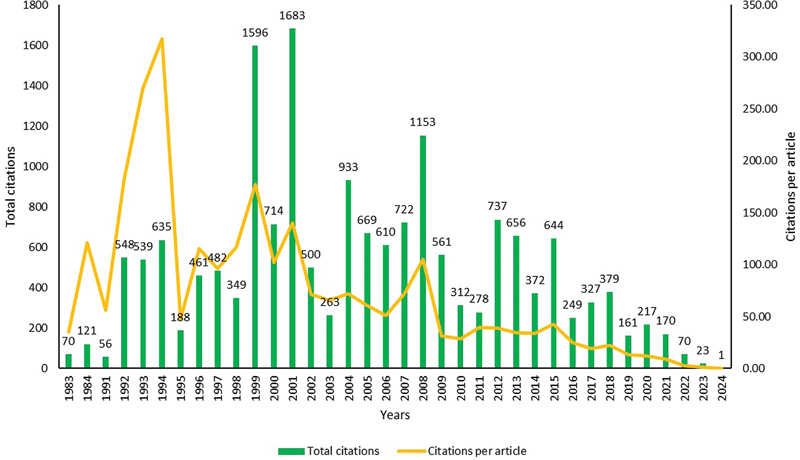
Trends of cited articles.

The descriptive outcomes of the selected articles are shown in Table 1 . Selected articles ( *n* = 366) were published in 157 journals between 1983 and 2024. The trend in article publication over time is shown in Fig. 2 , which also shows that the number of studies on QoL among HNC patients has increased continuously over time. The year 2022 had a maximum number ( *n* = 25; 6.83%) of articles published. The average number of publications was 10.17 articles per year. Since 1983, the growth rate has fluctuated; after 2016, the growth rate was significantly lower than the previous years. Out of 366 articles, the original articles were 318 (86.89%). The total number of citations of the selected articles was 17,449. The average citation per article was 47.67. Maximum citations (1,683) were in 2001. The trend of yearly total citations and yearly citations per article is shown in Fig. 3 .

**Table 1:** Descriptive statistics of selected articles

Variables	Outcomes
Total number of articles selected	366
Timespan	1983–2024
Total number of journals	157
Minimum–maximum article published in a year	1–25
Average articles published per year	10.17
Type of article	
• Original research	318 (86.89%)
• Review	48 (13.11%)
Total citations	17,449
Average citations per article	47.67
Average citations per year	484.69
Average citations per year per article	1.44
Average citations per article per year	1.32
Total number of countries where articles were published	52
Total authors	1,697
Articles per author	19.79
Authors per article	5.05
Author's keywords	414

### Countries and Institutions Analysis

**Fig. 4:**
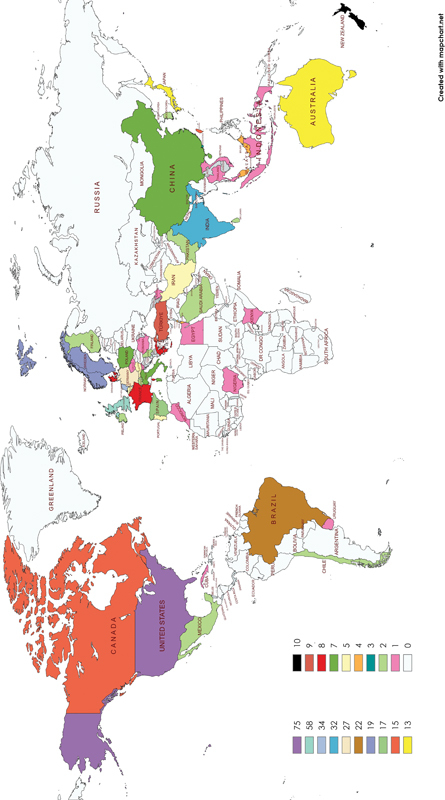
Countrywise publication of articles.

A total of 52 countries contributed to the research related to QoL among HNC patients. The United States ( *n* = 75; 20.49%) is a leading contributor to research, followed by the United Kingdom ( *n* = 58; 15.85%) and the Netherlands ( *n* = 34; 9.29%)- Supplementary Table S1 . The distribution is shown in Fig. 4 . The network mapping of collaboration between countries is shown in Supplementary Fig. S1 . 16 clusters have 214 links and a total link strength of 397. The degree of centrality was assessed to estimate the intensive collaboration. The United Kingdom has a centrality of 1.0, followed by the United States (0.89). Network mapping of collaboration between countries has a low density of 0.16. The density plot is shown in Supplementary Fig. S2 .

A total of 1,279 institutions have worked in the field of QoL among HNC patients. The regional Maxillofacial Unit, Liverpool University Hospital Aintree, United Kingdom, had the highest number of publications (4 articles). The Department of Otolaryngology–Head and Neck Surgery, University of Washington, United States; Department of Surgical Sciences, Faculty of Medicine, University of Bergen, Bergen, Norway; Division of Psychosocial Research and Epidemiology, Netherlands Cancer Institute, Amsterdam, Netherlands; and Faculty of Health and Social Sciences, Bergen University College, Bergen, Norway, published three articles each. Forty-eight institutes contributed 2 articles each, and 1,226 institutions had 1 article each.

### Authors Analysis

**Fig. 5:**
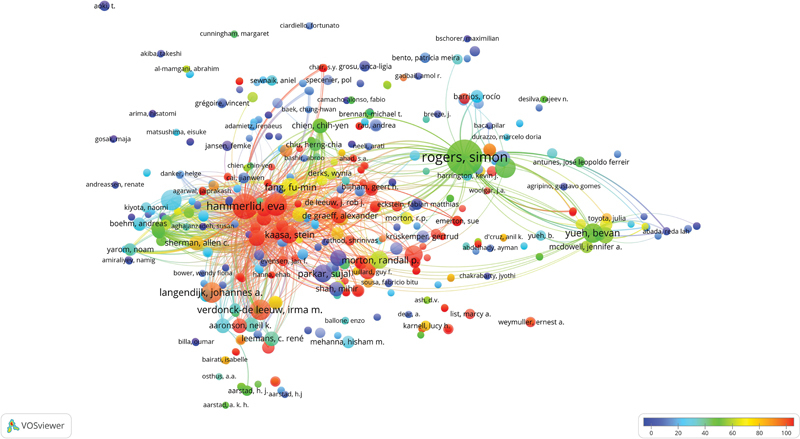
Network visualization map of potential authors. Cool colors represent fewer average citations than warm colors. Items-1,000; Clusters-8; Links-30,005; Total link strength-39,829.

A total of 1,697 authors contributed to the research on QoL among HNC patients. Among these, the top 10 potential authors who have published the most articles are shown in Table 2 . Roger Simon was the most potential author, having 24 articles with 1,198 citations and an average of 49.92 citations per article. The second top author was Hammerlid Eva, having 12 articles with 2,125 citations and an average of 177.08 citations per article. The network mapping of authors based on articles and average citations per article is shown in Fig. 5 . Each node on the map represents an author, and the circle size reflects the number of articles published by the authors. The network cluster was formed utilizing the colors of a rainbow, according to which cool colors demonstrate fewer average citations than warm colors. The network mapping displays 8 clusters, 30,005 links, and a total link strength of 39,829. Roger Simon had 525 links with a total link strength of 1,136, while Hammerlid Eva had 755 links with a total link strength of 2,198.

**Table 2:** Top ten potential authors based on the published articles and average citation per article

Rank ^a^	Author	Published articles	Total citations	Average citation per article
1st	Roger Simon	24	1,198	49.92
2nd	Hammerlid Eva	12	2,125	177.08
3rd	Bjordal Kristin	9	2,280	253.33
4th	Verdonck-De Leeuw Irma M.	8	772	96.50
4th	Langendijk Johannes A.	8	754	94.25
4th	Lowe D.	8	382	47.75
4th	Singer Susanne	8	219	27.38
8th	Yueh Bevan	7	355	50.71
9th	Kaasa Stein	6	2,090	348.33
9th	Ringash Jolie	6	603	100.50
9th	Fang Fu-Min	6	392	65.33
9th	Morton Randall P.	6	357	59.50
9th	Parkar Sujal	6	15	2.50

**Note:**^a^ In ranking, authors having equal number of articles were given similar ranks and the subsequent position in the rank was skipped.

The top 10 potential co-cited authors are shown in Supplementary Table S2 . Bjordal Kristin is the leading author, having a co-citation of 585, followed by Roger Simon with 519 co-citations. The network mapping of co-cited authors is displayed in Supplementary Fig. S3 . There were 5 clusters, 248,056 links, and a total link strength of 1,151,229. Bjordal Kristin (cluster 4; yellow), with a total link of 994 and with total link strength of 51,873, ranked first. Roger Simon (cluster 4; blue) with a total link of 993 and total link strength of 52,835 ranked second.

### Journals Analysis

**Fig. 6:**
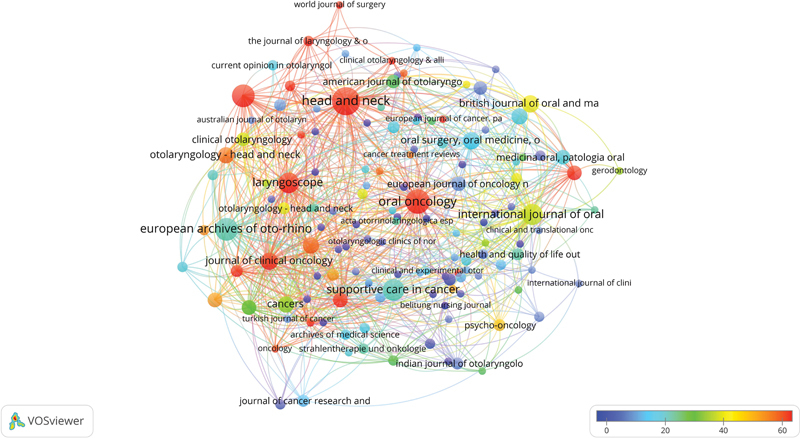
Network visualization map of impactful journals. Cool colors represent fewer average citations than warm colors. Items-155; Clusters-21; Links- 717; Total link strength-1,305.

In the research field of QoL among HNC patients, 155 scientific journals have published articles. The top 10 most impactful journals, along with their publisher and impact factor, are shown in Table 3 . The *Head and Neck* journal produced 24 (6.56%) articles, followed by the *Oral Oncology Journal* , contributing 17 (4.64%) articles. The network mapping of the journals is shown in Fig. 6 . The cold colors demonstrate fewer average citations than hot colors. The network mapping displays 21 clusters, 717 links, and a total link strength of 1,305. *Head and Neck* received the highest number of citations (901), followed by *Laryngoscope* (480) and *Archives of* *Otolaryngology-Head and Neck Surgery* (423) secured second and third rank respectively among co-cited journals ( Supplementary Table S3 ).

**Table 3:** Top ten most influential journals based on the published articles and average citation per article

Rank ^a^	Journal	Published articles	Total citations	Average citation per article	Publisher	Impact factor
1st	*Head and Neck*	24	2,627	109.46	John Wiley & Sons	2.9 (2022)
2nd	*Oral Oncology*	17	1,090	64.12	Elsevier	4.8 (2022)
3rd	*Archives of Otolaryngology-Head and Neck Surgery*	14	1,861	132.93	American Medical Association	8.96 (2021)
3rd	*European Archives of Oto-Rhino-Laryngology*	14	317	22.64	Springer Nature	5.3 2.6 (2022)
5th	*Supportive Care in Cancer*	13	287	22.08	Springer Nature	3.1 (2022)
6th	*International Journal of Oral and Maxillofacial Surgery*	12	445	37.08	Elsevier	2.4 (2022)
7th	*Laryngoscope*	11	1,184	107.64	John Wiley & Sons	2.6 (2022)
8th	*Journal of Clinical Oncology*	7	2,000	285.71	Wolters Kluwer Health	45.3 (2022)
8th	*British Journal of Oral and Maxillofacial Surgery*	7	280	40.00	Elsevier	1.8 (2022)
8th	*Oral Surgery, Oral Medicine, Oral Pathology, and Oral Radiology*	7	112	16.00	Elsevier	2.9 (2022)

**Note:**^a^ In ranking, journals having equal number of articles were given similar ranks and the subsequent position in the rank was skipped.

### Articles and Co-Cited Reference Analysis

**Fig. 7:**
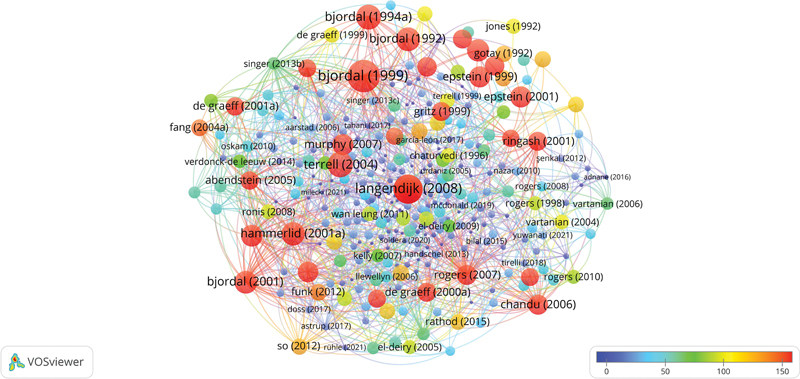
Network visualization map of top cited papers by authors. Cool colors represent fewer average citations than warm colors. Items-366; Clusters-51; Links- 1,355.

Table 4 displays the top 10 articles based on the citations they received since their publication. “Quality of life in head and neck cancer patients: validation of the European organization for research and treatment of cancer quality of life questionnaire-H and N 35” was the topmost article with 700 citations. This article was published by Bjordal et al. 16 in the *Journal of Clinical Oncology* in 1999. “Impact of late treatment-related toxicity on quality of life among patients with head and neck cancer treated with radiotherapy” by Langendijk et al. 17 ranked second, receiving 547 citations, and was published in the *Journal of Clinical Oncology* in 2008. Fig. 7 shows the network mapping of the top-cited articles by authors. Cool colors demonstrate fewer average citations than warm colors, amounting to 51 clusters with 1,355 links.

**Table 4:** Top ten most cited articles

Rank	Author	Title	Journal	Year	Citations
1st	Bjordal et al. 16	Quality of life in head and neck cancer patients: validation of the European organization for research and treatment of cancer quality of life questionnaire - H&N 35	*Journal of Clinical Oncology*	1999	700
2nd	Langendijk et al. 17	Impact of late treatment-related toxicity on quality of life among patients with head and neck cancer treated with radiotherapy	*Journal of Clinical Oncology*	2008	547
3rd	Hassan et al. 18	Assessment of quality of life in head and neck cancer patients	*Head and Neck*	1993	539
4th	Terrell et al. 19	Clinical predictors of quality of life in patients with head and neck cancer	*Archives of Otolaryngology-Head and Neck Surgery*	2004	377
5th	Bjordal et al. 20	Development of a European organization for research and treatment of cancer (EORTC) questionnaire module to be used in quality-of-life assessments in head and neck cancer patients	*Acta Oncologica*	1994	360
6th	Bjordal et al. 21	Psychometric validation of the EORTC core quality of life questionnaire, 30-item version and a diagnosis-specific module for head and neck cancer patients	*Acta Oncologica*	1992	320
7th	Hammerlid at al. 22	Health-related quality of life three years after diagnosis of head and neck cancer - A longitudinal study	*Head and Neck*	2001	300
8th	Bjordal et al. 23	A prospective study of quality of life in head and neck cancer patients. Part II: longitudinal data	*Laryngoscope*	2001	280
9th	Bjordal et al. 24	Quality of life in patients treated for head and neck cancer: a follow-up study 7 to 11 years after radiotherapy	*International Journal of Radiation Oncology, Biology Physics*	1994	275
10th	Hammerlid et al. 25	Health-related quality of life in long-term head and neck cancer survivors: A comparison with general population norms	*British Journal of Cancer*	2001	256

A co-citation relationship exists when more than two references are cited by more than one article simultaneously, and two or more connections are repeatedly cited references. 26 Supplementary Table S4 27 28 29 30 31 32 33 shows the top 10 co-cited references. The article published by Bjordal et al. titled “Psychological distress in head and neck cancer patients 7-11 years after curative treatment” received 22 co-citations. The network mapping of co-cited references is displayed in Supplementary Fig. S4 . Each node represents a cited article. The link between nodes represents the frequency with which a similar article is cited. The size of the node is proportional to the total number of citations. The network shows 9 clusters with 52,732 links, and a total link strength of 54,147. Nodes with similar colors show a cluster of related items.

### Keywords Analysis

**Fig. 8:**
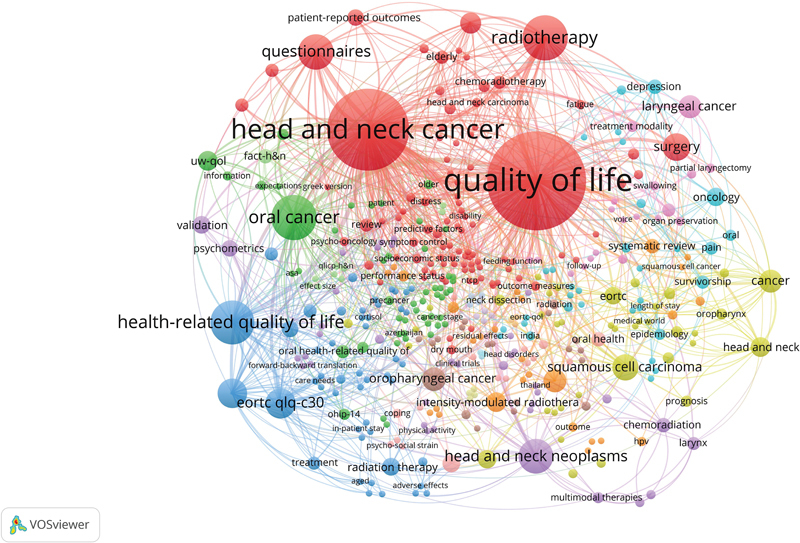
Network visualization map of author's keywords. Nodes with similar color represent a cluster of related items. Items-408; Clusters 16; Links-1,734; Total link strength-2,641.

A total of 408 keywords were identified. The network of co-occurrences of the author's keywords is shown in Fig. 8 . The most frequently occurring keyword was *Quality of life* ( *n* = 220) followed by *Head and neck cancer* ( *n* = 150), *Oral cancer* , and *Radiotherapy* ( *n* = 47). Network mapping shows that quality of life is the most central element in the network, with a centrality score of 1.0 displaying a most significant role and is highly linked to other elements within the network. The next most central element was head and neck cancer with a centrality score of 0.70. The network mapping of collaboration between keywords had a low density of 14.45. The density plot is shown in Supplementary Fig. S5 .

## Discussion

Assessing QoL among HNC patients is crucial as QoL assessments provide a comprehensive view of the patient's wellbeing, considering physical, emotional, social, and functional aspects. In addition, the impact of cancer treatments on QoL helps in evaluating the effectiveness of interventions beyond just survival rates. There are several QoL questionnaires specifically designed for assessing the impact of HNC. Examples of QoL questionnaires are the European Organization for Research and Treatment of Cancer Quality of Life Questionnaire Head and Neck 35 (EORTC QLQ-H&N35), Functional Assessment of Cancer Therapy-Head & Neck (FACT-H&N), University of Washington Quality of Life (UW-QoL), H&N Cancer QoL Scale, and Short Form Health Survey (SF-36). Each questionnaire has its own merits and demerits. For instance, EORTC QLQ-H&N-35 and FACT-H&N are often recommended for their comprehensive nature and focus on HNC-related issues. For clinical settings where time is limited, the UW-QOL may be preferable due to its brevity. In research settings, using a combination of disease-specific and general measures (e.g., EORTC QLQ-H&N35 along-side SF-36) can provide a more holistic view of a patient's quality of life. There is no single gold standard QOL instrument that is universally accepted as the most accurate. The choice of instrument often depends on the specific research objectives, study design, and patient population. Researchers should consider the psychometric properties (reliability, validity, responsiveness) of each instrument and select the one that best aligns with their study goals. The EORTC QLQ-H&N35 is validated and the most used questionnaire, hence, in this BA the articles related to EORTC QLQ-H&N35 were included.

The present study is the second BA of the articles on QoL among HNC patients. It includes 366 articles written in English, published in 157 journals by 1,279 institutions in 52 countries. The data were extracted from the Scopus database. The Scopus database was selected because: 1) Scopus is a large database containing 27,950 active peer-reviewed journals with 20.54 million open access articles, 34 2) Scopus allows for searches using terms in titles, titles/abstracts, the name of the journal, the name of the author, or affiliation, and 3) the export of data from Scopus is simple to carry-out. The VOSviewer software was used for the BA as it is user-friendly and supports a wide range of sources, allowing comprehensive construction, visualization, and analysis of bibliometric maps. In this section, the common bibliometric indicators such as trends in research publications, the most potential researchers, the top research publications, top productive journals, countries, and research institutions, and frequently occurring keywords are discussed as follows.

The annual trends showed a mixed pattern of increase and decrease in number of publications. During the early years, the number of publications was too low. However, publications gradually rose from 2000 onward. Nearly half ( *n* = 169; 46.17%) of the articles related to QoL among HNC patients were published in the last decade. Until May 2024, 5 publications were retrieved. Due to the advancement in the management of HNC, the survival rates have improved, and more patients are living longer with the disease, with a greater emphasis on enhancing their overall QoL during and after treatment. Healthcare professionals, researchers, and policymakers are increasingly focusing on holistic care that considers the physical functioning, psychological, and social wellbeing of the patients as well. Thus, the importance of assessing and improving the QoL of patients with HNC is expected to grow in numbers in the coming years.

The United States demonstrates its dominance in QoL research among HNC patients with a contribution of 75 articles and collaboration with other countries. This finding was in line with a previous BA. 13 Along with the United States, other European countries, such as the United Kingdom and the Netherlands, have a good number of articles, showing a strong collaboration for future study. High-income countries had more vigorous research capacity in QoL research as they had several specialized research institutions, entrenched infrastructure and data systems, and funding for research. 35 The network mapping of collaboration among countries shows the United States and the United Kingdom expanded their partnership with Asian countries (red cluster). Green clusters showed collaboration between European countries. This might be due to the cross-cultural studies within the geographic region. Such studies will help to assess QoL across different cultures by using culturally sensitive and valid tools informing health care policies and effective interventions. 36

There was a heterogeneous contribution from different institutes. Of 1,279 institutes, Liverpool University Hospitals, United Kingdom, contributed 4 articles, while 3 articles each were published from the University of Washington, United States; Faculty of Medicine, University of Bergen, Norway; Netherlands Cancer Institute, Netherlands; and Faculty of Health and Social Sciences, University of Bergen, Norway. Most of the institutes (95.86%) contributed with only one article. This suggests that the research groups and institutions are scattered and have low scientific evidence in QoL research, and hence the collaboration must be increased.

Roger Simon contributed the most in terms of publications in this field. Furthermore, Hammerlid Eva and Bjordal Kristine were the top three potential contributors to this research field. These authors are all renowned researchers in the field of QoL among HNC patients. One of their notable works is the development and validation of the EORTC-QLQ H&N35. 16 The top 10 co-cited authors with at least 165 co-citations had a significant contribution.

*Head and Neck* journal (24) ranks first considering total publications, followed by *Oral Oncology* (17), and *Archives of Otolaryngology-Head and Neck Surgery* and *European Archives of Oto-Rhino-Laryngology* , (14/each). These interdisciplinary journals publish research on the etiopathogenesis, epidemiology, diagnosis, prevention, and management of head and neck diseases. Publishing in such journals increases the visibility of the research, leading to more citations and recognition of the researcher in medical oncology, head and neck surgery and radiotherapy, maxillo-facial surgeons, dental professionals, and many others engaged in these fields. The results of co-citation journals will assist future researchers in selecting journals and submitting their research related to QoL among HNC patients for publication.

The top 10 articles published in the mid-90s and early 20s are considered landmark studies in the field of QoL for HNC patients. The article titled “Quality of life in head and neck cancer patients: validation of the European organization for research and treatment of cancer quality of life questionnaire-H and N 35” published by Bjordal et al. 16 was the topmost article, with 700 citations. The article was published in the *Journal of Clinical Oncology* in 1999. This is a classical study involving 500 patients from Norway, Sweden, and the Netherlands to define the scales and test the validity, reliability, and sensitivity of the head and neck-specific questionnaire EORTC QLQ-H&N35 to assess the QoL of HNC patients combined with the general questionnaire EORTC QLQ-C30. The co-citation of references showed a relatively balanced publication of the articles during the mid-90s and early-20s, indicating that attention and interest from the researchers in this field had been sustained. Co-citation analysis can provide a wealth of information, warranting future researchers to gain in-depth insight into QoL among HNC patients.

The 3 frequently occurring author's keywords were *quality of life* (250), *head and neck cancer* (150), *oral cancer* , and *radiotherapy* (47). Considering these keywords, more research needs to be conducted, as these words form the core of this field. The sharing of keywords with similar research is represented by linking lines. For example, *quality of life* (in red) is part of the head and neck cancer, radiotherapy, questionnaire, surgery, etc. The width of the line joining keywords to nodes is reciprocally related. Many authors' keywords were only used once or twice, which indicated a lack of continuity in QoL research and a wide disparity in research focus. 37

Recently, BA has gained great popularity in the research field. Researchers use BA to know the trends of articles, journal performance, collaboration patterns between countries and institutions, co-authorship, and keywords used. 12 Even though the network mapping was performed meticulously, the findings should be interpreted with caution as the present study has a few limitations. The first one is that the BA was performed only on papers published in the Scopus database; thus, articles indexed in other databases were not considered; therefore, the sample used in our study is not representative of the sum of research in the fields of QoL among HNC patients. However, Scopus is the largest database and may contribute to achieving reliable results. The second limitation is that relevant articles published in languages other than English language were not included, resulting in linguistic bias. The third limitation concerns self-citations, as this was not considered while performing citation analysis. Self-citation may pose a bias in the number of citations for authors, journals, and countries. Finally, the fourth limitation is that older articles can have significantly high citation numbers, due to their prolonged presence in the open domain.

## Conclusion

The present paper provides a comprehensive summary of trends in QoL research in HNC patients. This BA revealed that there is significant and increasing development in this field of research. There were disparities in scientific work country-wise and a lack of collaboration between the institutes. These disparities may be due to the economic level, healthcare systems, and academic collaboration models of respective countries and institutions. The current BA identifies the potential researchers and institutions in the field of QoL among HNC, which further helps in understanding the major contributors and potential collaborators for future studies. Considering all the strengths and limitations of the current study, it provides researchers and policymakers with baseline data to frame and implement interventional strategies to improve the survival and enhance the overall QoL of HNC patients.
