# Supplementary material for: Head and Neck Cancer Patients' Quality of Life: A Bibliometric Analysis Using Network Visualization Mapping
Source: Int Arch Otorhinolaryngol. 2025 Sep 19;29(3):1–13. doi: 10.1055/s-0045-1809665 (PMC12449100; doi:10.1055/s-0045-1809665)
Supplement: Supplementary file 1 — Supplementary Material [file 10-1055-s-0045-1809665-s241799.pdf]

Supplementary Table S1 Top ten countries based on the published articles

Rank[a]	Country	Published articles	Total citations	Centrality
1st	United States	75	5227	0.89
2nd	United Kingdom	58	3359	1.0
3rd	Netherlands	34	2862	0.79
4th	India	32	680	0.25
5th	Germany	27	625	0.79
6th	Brazil	22	546	0.64
7th	Sweden	19	2566	0.71
7th	Norway	19	2308	0.68
9th	Spain	17	590	0.61
10th	Taiwan	15	645	0.64
10th	Canada	15	1269	0.07

[a]In ranking, authors having equal number of articles were given similar ranks and the subsequent position in the rank was skipped.

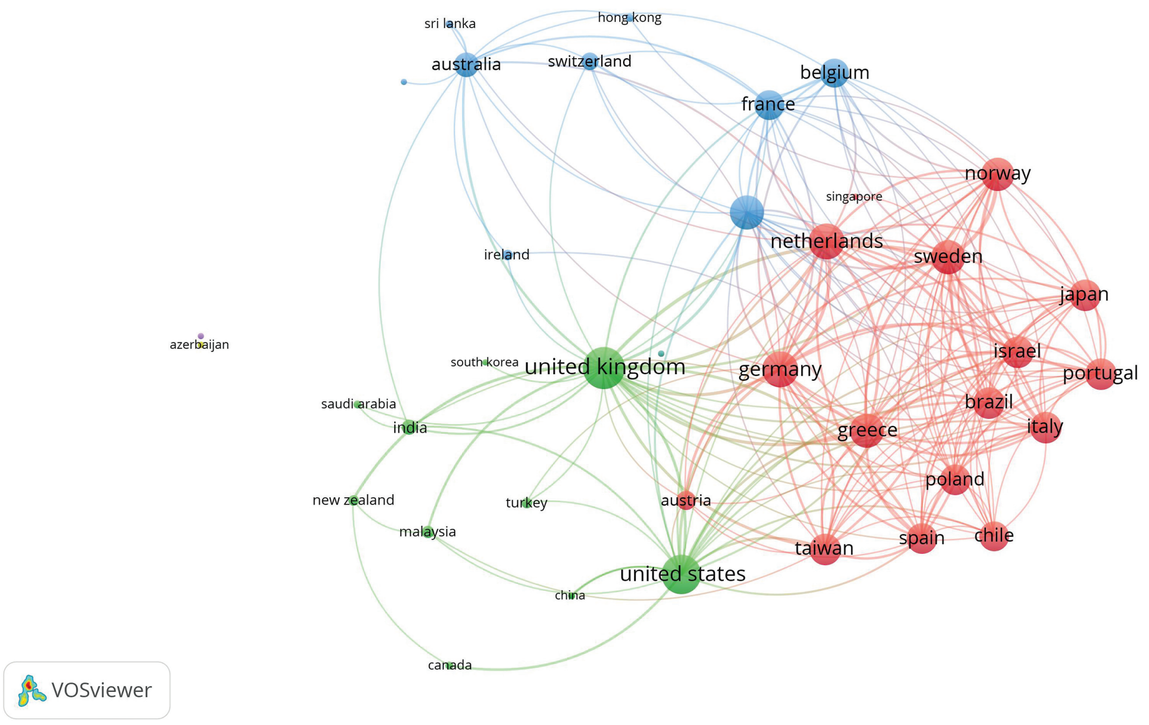

Supplementary Fig. S1 Network visualization map of collaboration between countries. Nodes with similar color represent a cluster of related items. Items-52; Clusters-16; Links- 214; Total link strength-397.

 © 2025. The Author(s).

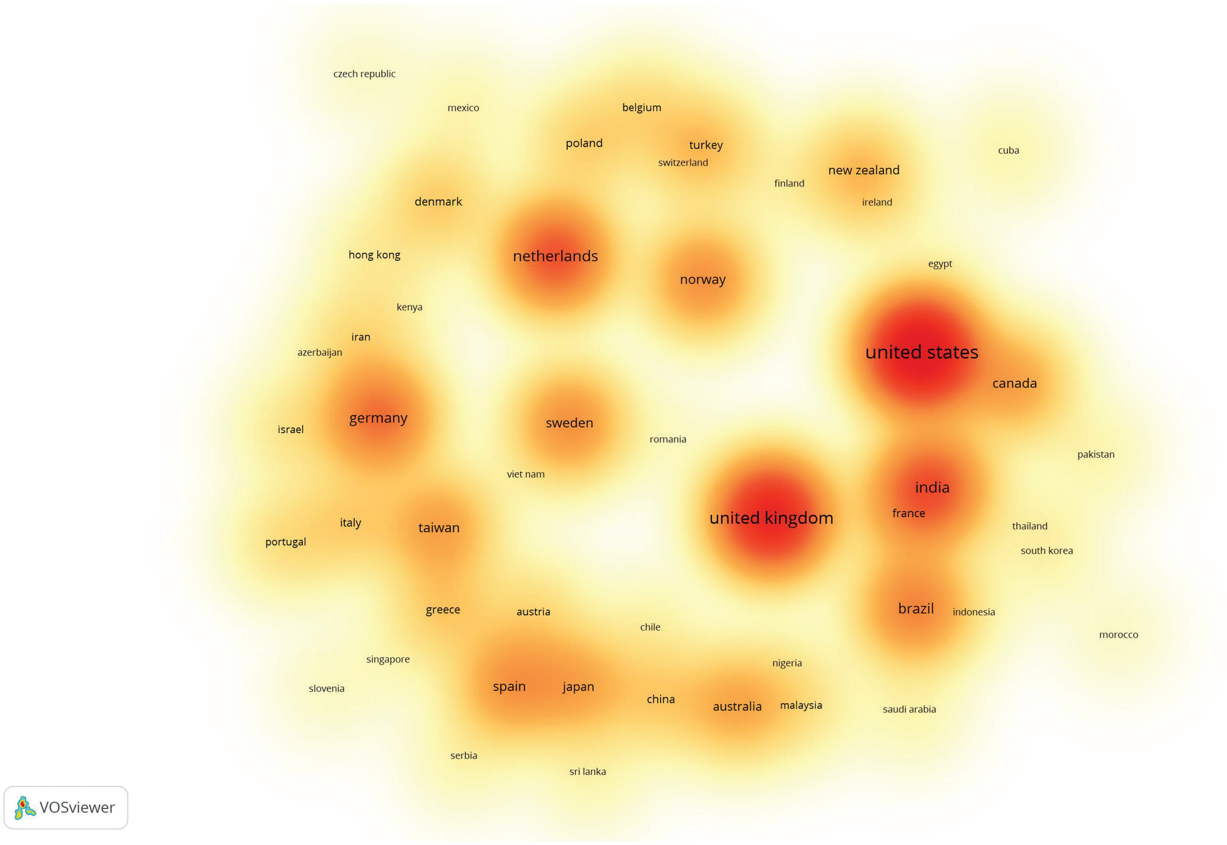

Supplementary Fig. S2 Density map of collaboration between countries. Higher density is denoted by warm colors.

Supplementary Table S2 Top ten co-cited authors based average total citation

Rank[a]	Author	Total co-citations
1st	Bjordal Kristin	585
2nd	Rogers Simon	519
3rd	Hammerlid Eva	438
4th	Lowe D.	357
5th	Ahlner-Elmqvist M.	299
6th	Aaronson N.K.	274
7th	De Graeff A.	222
8th	Kaasa Stein	219
9th	Morton Randall P.	184
10th	Terrell Jeffrey E.	165

 © 2025. The Author(s).

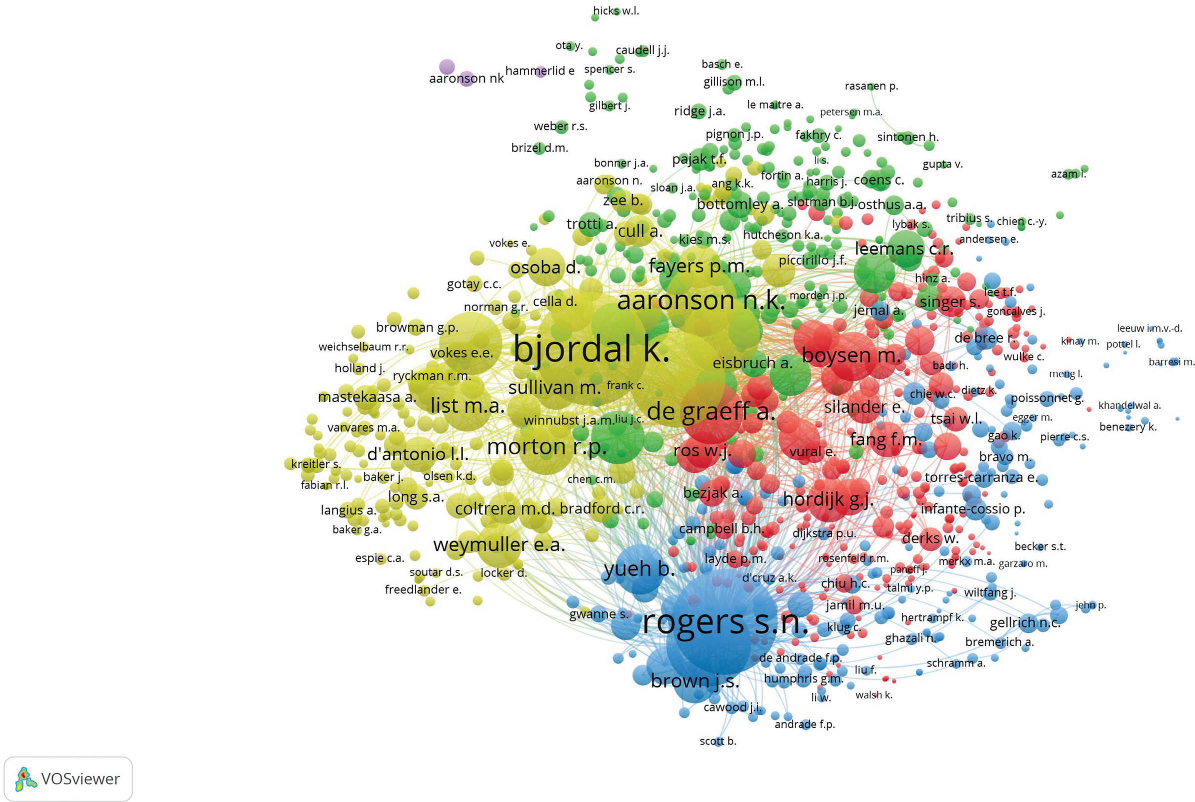

Supplementary Fig. S3 Network visualization map of co-cited authors. Nodes with similar color represent a cluster of related items. Items-1,000, Clusters-5; Links- 248,056; Total link strength-1,151,229.

Supplementary Table S3 Top ten co-cited journals based average total citation

Rank	Cited Journals	Total citations
1st	Head and Neck	901
2nd	Laryngoscope	480
3rd	Archives Of Otolaryngology - Head and Neck Surgery	423
4th	Journal Of Clinical Oncology	399
5th	Oral Oncology	387
6th	Cancer	329
7th	International Journal of Radiation Oncology, Biology Physics	288
8th	Quality of Life Research	203
9th	European Journal of Cancer	161
10th	British Journal of Cancer	143

 © 2025. The Author(s).

Supplementary Table S4 Top ten most co-cited references

Rank[a]	Author	Title	Journal	Year	Co-Citations
1st	Bjordal K et al[27]	Psychological distress in head and neck cancer patients 7–11 years after curative treatment	British Journal of Cancer	1995	22
2nd	Hassan SJ et al[18]	Assessment of quality of life in head and neck cancer patients	Head and Neck	1993	21
3rd	Bjordal K et al[21]	Psychometric validation of the EORTC core quality of life questionnaire, 30-item version and a diagnosis-specific module for head and neck cancer patients	Acta Oncologica	1992	20
4th	Hammerlid E et al[25]	Health-related quality of life in long-term head and neck cancer survivors: A comparison with general population norms	British Journal of Cancer	2001	16
5th	Aaronson NK et al[28]	The European organization for research and treatment of cancer QLQ-C30: a quality-of-life instrument for use in international clinical trials in oncology	Journal of the National Cancer Institute	1993	15
6th	Chandu A et al[29]	Health-related quality of life in oral cancer: a review	Journal of Oral Maxillofacial Surgery	2006	14
6th	Ringash J at al[30]	Structured review of quality of life instruments for head and neck cancer patients	Head and neck	2001	14
8th	List MA et al[31]	Longitudinal assessment of quality of life in laryngeal cancer patients	Head and neck	1996	13
8th	Tschudi D et al[32]	Quality of life after different treatment modalities for carcinoma of the oropharynx	Laryngoscope	2003	13
10th	Bjordal K et al[16]	Quality of life in head and neck cancer patients: validation of the European organization for research and treatment of cancer quality of life questionnaire- H&N 35	Journal of Clinical Oncology	1999	12
10th	Bjordal K et al[24]	Quality of life in patients treated for head and neck cancer: a follow-up study 7 to 11 years after radiotherapy	International Journal of Radiation Oncology, Biology Physics	1994	12
10th	King MT[33]	The interpretation of scores from the EORTC quality of life questionnaire QLQ-C30	Quality of Life Research	1996	12

[a]In ranking, authors having equal number of articles were given similar ranks and the subsequent position in the rank was skipped.

 © 2025. The Author(s).

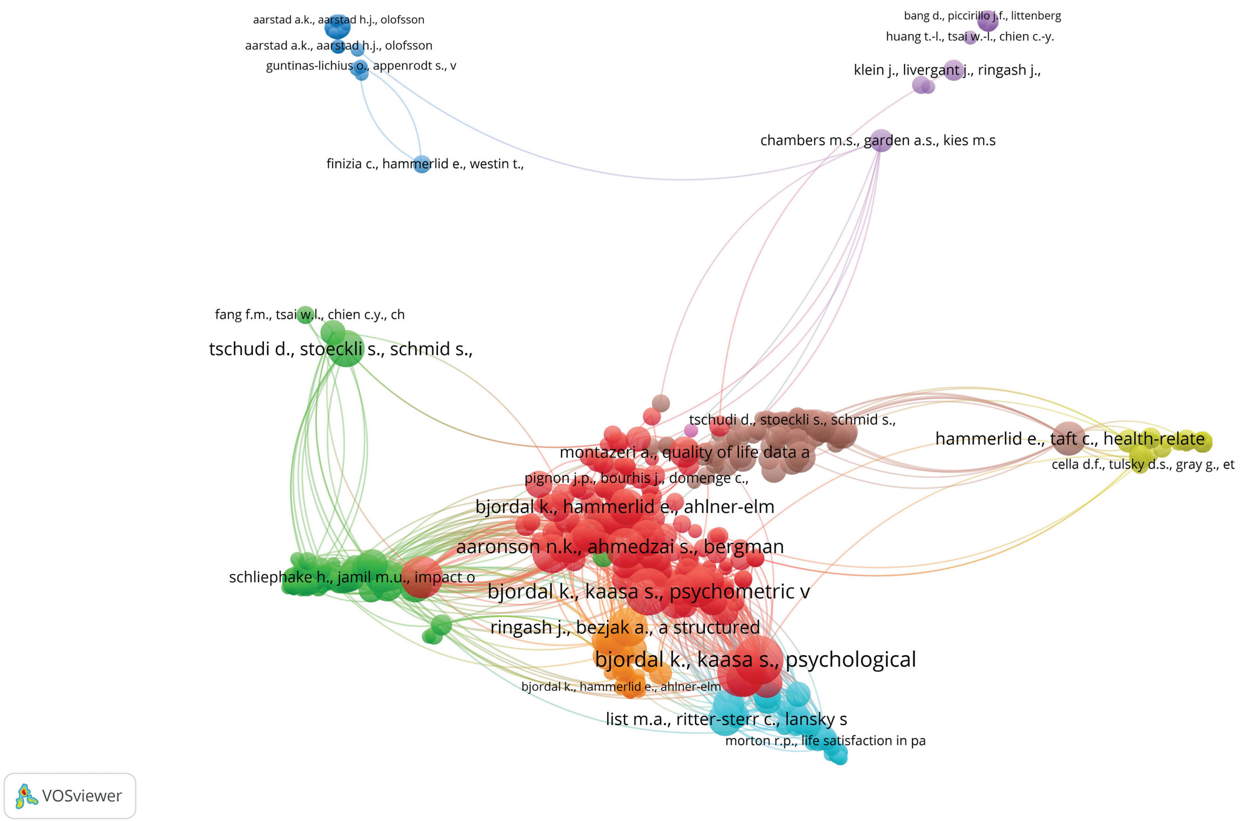

Supplementary Fig. S4 Network visualization map of top co-cited references. Nodes with similar color represent a cluster of related items. Items-1,000; Clusters 9; Links-52,732; Total link strength-54,147.

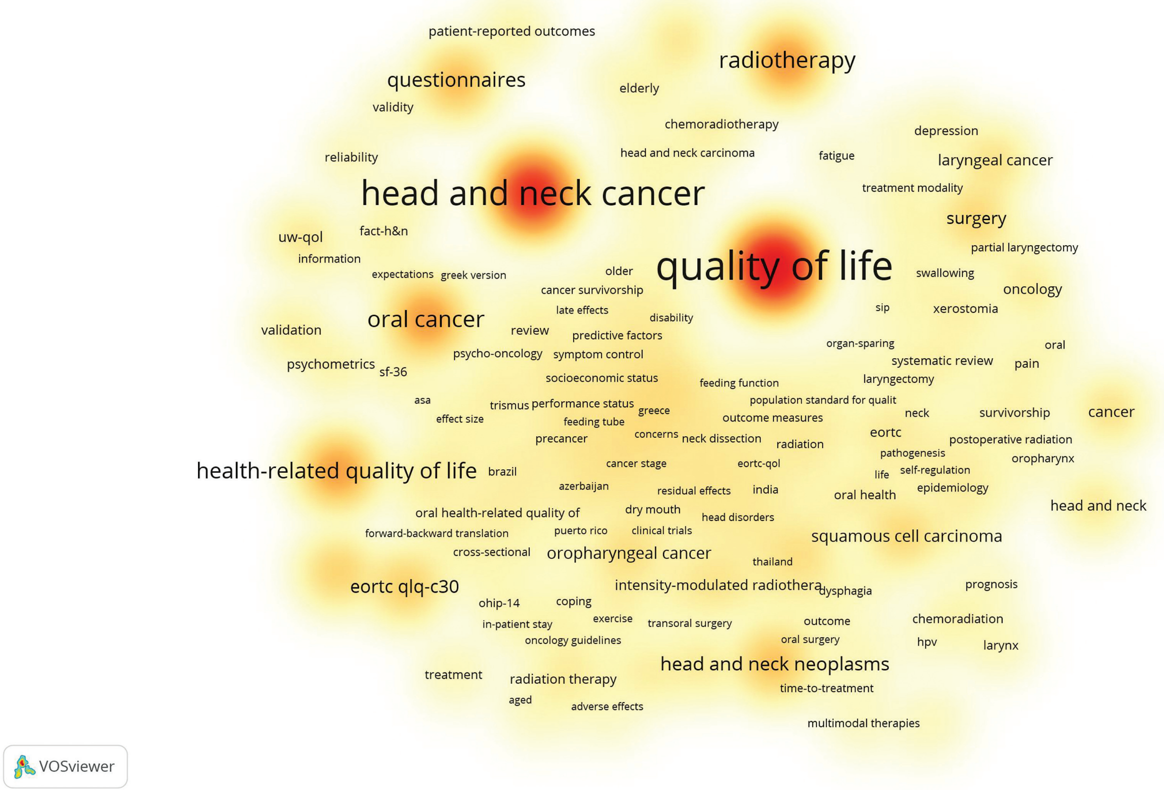

Supplementary Fig. S5 Density map of collaboration between keywords. Higher density is denoted by warm colors.

 © 2025. The Author(s).
